# Discovery and Characterization Small Molecule Modulators of Striatal‐Enriched Protein Tyrosine Phosphatase (STEP) for Alzheimer’s Disease Therapeutics

**DOI:** 10.1002/alz.083572

**Published:** 2025-01-09

**Authors:** Jiaqian Wu, Ye Na Han, Nicholas Cosford, Lutz Tautz

**Affiliations:** ^1^ Sanford burnham prebys medical discovery institute, San Diego, CA USA

## Abstract

**Background:**

A pathological hallmark of Alzheimer’s disease (AD) is the accumulation of amyloid‐beta peptide (Aß). Potential treatments targeting Aß production such as γ‐secretase inhibitors have had limited success. A promising alternative approach involves addressing early synaptic dysfunction by modulating molecules like striatal‐enriched protein tyrosine phosphatase (STEP), whose levels and activity are upregulated by Aß. The current model of STEP function is that it opposes the development of synaptic strengthening, and that high levels of STEP contribute to the cognitive deficits in AD. Importantly, genetic reduction or chemical inhibition of STEP reverses the cognitive and cellular deficits in an AD mouse model, validating STEP as a drug target in AD.

**Method:**

Using ^19^F nuclear magnetic resonance (NMR) spectroscopy, a library of 1,000 fluorine‐containing fragments was screened to identify novel STEP binders. Initial hits were validated using a combination of biophysical binding assays including ^19^F NMR, 1D WaterLOGSY NMR, microscale thermophoresis (MST), and isothermal titration calorimetry (ITC). Based on a potent and selective fragment, STEP‐targeted protein degraders were designed, synthesized, and characterized in cellular experiments.

**Result:**

Several potent and selective fragments were identified and validated. Based on the most potent binder, five proteolysis targeting chimera (PROTAC) molecules were initially synthesized and characterized. These compounds demonstrated degradation of STEP in SH‐SY5Y neuroblastoma cells. One STEP PROTAC was able to penetrate the blood‐brain barrier and showed potent degradation efficacy *in vivo*.

**Conclusion:**

Our fragment‐based discovery platform to identify novel STEP binders for the development of specific degrader molecules has yielded first‐in‐class STEP PROTACs. By targeting STEP and thus synaptic dysfunction, our research could provide valuable insights into the early stages of AD pathogenesis and contribute to the development of effective therapeutic strategies for this devastating disease.

